# Repurposed metformin–apigenin combination therapy for metabolic dysfunction-associated steatohepatitis: from integrative multi-omics discovery to *in vivo* validation

**DOI:** 10.3389/fphar.2026.1857676

**Published:** 2026-09-04

**Authors:** Kanglong Zhang, Yihua Chen, Linwei Ran, Weizhao Tong, Lu Dai, Zhen Yang, Qi Chen, Jie Zheng, Guoxin Hu

**Affiliations:** 1 Department of Infectious Diseases, Peking University Shenzhen Hospital, Shenzhen Peking University-The Hong Kong University of Science and Technology Medical Center, Shenzhen, Guangdong, China; 2 Department of Infectious Diseases, Peking University Shenzhen Hospital, Shenzhen, Guangdong, China; 3 Department of Ultrasound, Shenzhen Key Laboratory for Drug Addiction and Medication Safety, Institute of Ultrasonic Medicine, Peking University Shenzhen Hospital, Shenzhen Peking University- The Hong Kong University of Science and Technology Medical Center, Shenzhen, Guangdong, China; 4 Department of Traditional Chinese Medicine, Peking University Shenzhen Hospital, Shenzhen, Guangdong, China; 5 Shenzhen Key Laboratory of Immunity and Inflammatory Diseases, Shenzhen, Guangdong, China

**Keywords:** drug repurposing, lipid dysregulation and inflammation, metabolic dysfunction-associated steatohepatitis, metformin–apigenin combination therapy, multi-omics

## Abstract

**Background:**

Metabolic dysfunction-associated steatohepatitis (MASH) is a chronic liver disease. Although recent drugs have been approved, the complex pathophysiology of MASH limits monotherapy efficacy and generates considerable inter-patient heterogeneity. The purpose of this research was to identify new molecular targets and develop a rational combination therapeutic approach for MASH.

**Methods:**

To determine the molecular signatures of MASH mouse models and healthy controls, we conducted bulk RNA sequencing on liver tissues and then analyzed the data with a variety of Gene Ontology, Kyoto Encyclopedia of Genes and Genomes pathway enrichment, weighted gene co-expression network analysis, and so on. The role of lipidomic profiling was to determine the relation between genes and lipids. The human MASH liver specimens were used to test clinical relevance. Causal relationships were measured using Mendelian randomization (MR). Direct interaction between the drugs and the target was predicted by molecular docking and molecular dynamics simulations. The efficacy of metformin and apigenin as single therapy and in combination was assessed *in vivo* using MASH mice.

**Results:**

We discovered a five-gene signature (Gpd2, Cybb, Clec12a, Ifi211, and Tifab), which was highly expressed by MASH and was strongly connected to lipid metabolism and inflammatory events. Integrative lipidomic correlation analysis showed that these genes were specifically linked with certain lipotoxic species. Molecular docking and molecular dynamics simulation studies have shown that metformin and apigenin, respectively, bind to Gpd2 and Cybb directly. Both agents alleviated the MASH pathology, and combined therapy was more effective than monotherapy.

**Conclusion:**

This study provides a new five-gene signature transcending hepatic lipid dysregulation and inflammation associated with MASH. The demonstration that metformin targets Gpd2 and apigenin targets Cybb provides a mechanistic basis for combination therapy, which exhibited superior efficacy over monotherapy. This repurposed oral drug combination offers a promising multi-target strategy for MASH treatment.

## 1 Introduction

Metabolic dysfunction-associated steatohepatitis (MASH) represents a severe and progressive form of metabolic dysfunction-associated steatotic liver disease (MASLD), which is pathologically characterized by hepatic steatosis, lobular inflammation, hepatocellular ballooning, and progressive fibrosis (Rinella et al., 2023). Affecting approximately 3%–7% of the global population, it continues to escalate alongside the worldwide epidemics of obesity and type 2 diabetes mellitus (T2MD), establishing MASH as a major etiology of chronic liver disease, cirrhosis, hepatocellular carcinoma (HCC), and a mounting global health threat (Castera et al., 2025; Younossi et al., 2026). Lifestyle modifications remain the cornerstone of MASLD/MASH management (Tilg et al., 2026). However, long-term adherence is poor, and frequent weight regain is common (European Association for the Study, 2024). Consequently, sustained weight reduction of ≥10%, which is necessary for histological improvement, is achieved and maintained by only a minority of patients (Malespin et al., 2022; European Association for the Study of the et al., 2024; Younossi et al., 2025). Recent breakthroughs have led to the conditional approval of resmetirom (Chen et al., 2025) and semaglutide (Sanyal et al., 2025) by the US Food and Drug Administration for noncirrhotic MASH with moderate-to-severe fibrosis (stages F2–F3). However, emerging clinical evidence indicates that, despite achieving fibrosis improvement in a proportion of patients compared with placebo (Harrison et al., 2024; Sanyal et al., 2025), these monotherapies demonstrate limited overall absolute benefit and considerable heterogeneity in treatment responses across patient populations, particularly among those with versus without concomitant T2MD (Musso et al., 2026). Consequently, significant unmet medical needs persist, underscoring the critical importance of developing novel therapeutic strategies.

The classical two-hit theory of disease is now considered an insufficient account for the profound inter-patient heterogeneity of MASH (Buzzetti et al., 2016). MASH is a multifactorial condition coordinated by a complicated combination of genetic predisposition, epigenetic alterations, intestinal dysbiosis, insulin resistance, maladjusted lipid metabolism, oxidative stress, endoplasmic reticulum stress, and sustained inflammatory signaling (Buzzetti et al., 2016; Pourteymour et al., 2023; Kang et al., 2025; Li Y. et al., 2025; Steinberg et al., 2025; Wu et al., 2025). This pathobiological complexity indicates that the mono-therapeutic interventions that affect only one molecular node will not be effective in heterogeneous MASH populations. Due to the multifactorial characteristics of the disease and the low response rates that are observed with single-agent therapy, an increased acceptance of the idea that combination regimens can have synergistic effects is increasing. These regimens would also simultaneously address several disease-promoting pathways, such as dysmetabolism, inflammatory pathways, and fibrosis (Do et al., 2025; Newsome and Loomba, 2025). Nevertheless, initial clinical studies on combination therapies have had mixed outcomes, and it remains unclear how the best combinations of drugs can be rationally designed. Thus, the identification of novel, mechanism-based combination regimens, particularly those repurposing existing drugs with established safety profiles, is essential for current MASH therapeutics.

Our objective in this study was not only to develop a comprehensive catalog of major molecular pathways that are involved in the pathogenesis of MASH through the combination of genomic and lipidomic analyses but also to use our results to develop a novel multi-target therapeutic methodology. Bulk RNA-sequencing liver tissues from MASH-model mice and healthy controls revealed a typical five-gene signature (Gpd2, Cybb, Clec12a, Ifi211, and Tifab), which was significantly elevated in MASH liver tissues. Extensive bioinformatics systematically associated these genes with lipid metabolism and inflammatory reactions. Later lipidomic analysis indicated that this gene signature was correlated with certain lipid species, including diacylglycerols and phospholipids. This enabled us to develop a gene–lipid regulatory network. We verified that four of these genes (which were Gpd2, Cybb, Clec12a, and Ifi211) are aberrantly overexpressed in human MASH liver specimens. Although the studies of Mendelian randomization (MR) did not show a direct relationship between the genes and MASLD, it is a probable indication of complicated downstream effects or environmental interference, which indicates a requirement to validate the functions of these networks and target them therapeutically instead of merely modulating these genes.

Structure-based molecular docking predicted favorable binding potential between the classic antidiabetic agent metformin and Gpd2 and between the natural flavonoid apigenin and Cybb. Finally, *in vivo* tests confirmed that metformin and apigenin effectively ameliorate key pathological features of MASH, with combined therapy demonstrating superior efficacy compared with monotherapy. Collectively, this study not only introduces the first gene signature intimately connected to the lipid-metabolic and inflammatory networks of MASH but also provides a novel combinatorial pharmacologic regimen for multi-target intervention, thereby opening new avenues for precision therapy of MASH.

## 2 Materials and methods

### 2.1 Animal model, sample collection, and ethics statement

Male C57BL/6J mice aged 6 weeks (19–22 g, SPF grade) were acquired in the Vital River Laboratory Animal Technology Co., Ltd (Beijing, China) and were kept in a 12-h light/dark cycle at 25 °C and 40–60 per cent humidity. One week after acclimatization, the mice were randomly grouped into a normal chow diet group and a high-fat, high-cholesterol diet group fed the Amylin Liver NASH (AMLN) purified rodent diet (Dyets, China, Cat. No. AMLN) for 24 weeks. The diet contains 40% fat calories (including 2% cholesterol by wt), 20% protein calories, and 40% carbohydrate calories (including 22% fructose by wt). At the end point, the livers were immediately removed and then cut into small pieces weighing no more than 50 mg. One sample was affixed in 4% paraformaldehyde to be tested later using hematoxylin and eosin (H&E) and Sirius Red staining. The other two samples were used to test using oil red O staining and bulk RNA and lipidomics sequencing. To determine alanine aminotransferase (ALT), aspartate aminotransferase (AST), triglycerides (TGs), total cholesterol (TCHO), high-density lipoprotein (HDL), and low-density lipoprotein (LDL), blood samples were collected. The Experimental Animal Welfare Ethics Committee of Shenzhen Peking University–The Hong Kong University of Science and Technology Medical Center approved all live animal research in accordance with the Experimental Animal Welfare Act (Experiment No. 2024–160).

### 2.2 RNA extraction, sequencing, and data filtering

Total RNA was extracted from liver tissues using TRIzol (Invitrogen, Carlsbad, CA, United States of America), followed by quality assessment and quantification with an Agilent 2100 Bioanalyzer (Agilent, CA, United States of America). mRNA libraries were constructed using the BGI Optimal dual-module kit (BGI-Shenzhen, China) according to the following workflow: mRNA enrichment and fragmentation with oligo (dT) beads, reverse transcription into double-stranded cDNA using random primers, end repair, adapter ligation, PCR amplification, circularization, and digestion. DNA nanoballs (DNBs) were prepared from the resulting libraries and sequenced on the DNBSEQ-T7 platform (BGI-Shenzhen, China) in paired-end 150 (PE150) mode. Raw sequencing data were processed with SOAPnuke (v2.2.1) (Li et al., 2008) to remove adapter-contaminated reads, low-quality reads (≥20% bases with Phred score ≤15), and reads with >5% undetermined nucleotides (N), yielding clean data for downstream bioinformatic analyses. The raw data have been deposited in the CNGB Nucleotide Sequence Archive (CNSA) under accession number CNP0008322.

### 2.3 Differential gene expression, GO/KEGG enrichment, and WGCNA analysis

The distribution of samples was assessed using principal component analysis (PCA) with the stats package used in R (v 3.6.0). DEGs were determined between the MASH and normal control groups using DESeq2 (v1.42.1) (Love et al., 2014) with an adjusted P-value <0.05 and |log2FC| > 0.585 (corresponding to a 1.5-fold change) as the significance thresholds, which were intentionally selected based on previous MASH studies (Latif et al., 2022). The visualization of DEGs was conducted using volcano plots and heatmaps. ClusterProfiler (v4.10.0) (Yu et al., 2012) was used to conduct functional enrichment analyses, such as Gene Ontology (GO) (Ashburner et al., 2000) (Biological Process, Cellular Component, and Molecular Function) and Kyoto Encyclopedia of Genes and Genomes (KEGG) pathways (Kanehisa and Goto, 2000). Statistically significant enriched terms whose P-value was adjusted below 0.05 were taken as significant, and representative terms were taken for visualization. Weighted gene co-expression network analysis (WGCNA) R package (Version 1.61) was used as part of the analysis of the top 5,000 genes ranked by their median absolute deviation (MAD) values (Langfelder and Horvath, 2008). The resulting hub genes that were identified using WGCNA were intersected with the already identified DEGs to obtain candidate genes to be used in further analyses.

### 2.4 Biomarkers identification, validation, and nomogram development

Candidate genes were prioritized by intersecting the feature selection results from least absolute shrinkage and selection operator (LASSO) regression (glmnet v4.1.3) (Gao et al., 2010) and the Boruta algorithm (v8.0.0 https://cran.r-project.org/web/packages/Boruta/). Hub genes were subsequently subjected to the Wilcoxon rank-sum test between MASH and control groups and evaluated by receiver operating characteristic (ROC) analysis using the pROC package (v1.18.0) (Robin et al., 2011). Genes with area under the curve (AUC) > 0.7 and statistically significant differences (p < 0.05) were selected as biomarkers. Spearman correlation analysis was finally performed among the identified biomarkers. A nomogram was constructed based on biomarker expression in the training set using the rms package (v6.7-0) (Fang et al., 2024), with diagnostic performance assessed by calibration and ROC curve analyses.

### 2.5 GSEA and immune infiltration analysis

In the case of each biomarker, disease samples were dichotomized by high and low expression levels, using median levels of expression. Gene Set Enrichment Analysis (GSEA) was performed through the clusterProfiler package (Yu et al., 2012) with KEGG gene sets (FDR < 0.05) to determine pathways that were highly correlated with the biomarker expression. The Cell-type Identification by Estimating Relative Subsets of RNA Transcripts (CIBERSORT) algorithm was used to deconvolute the immune cell composition across samples (Chen et al., 2018; Kawada et al., 2021). Immunological disparities of disease and control groups were plotted using a box plot and assessed using the Wilcoxon rank-sum test. The Pearson correlation analysis was then used to evaluate the correlation between immune cells that were significantly different (P < 0.05) and biomarkers, and the findings were presented in the form of a heatmap.

### 2.6 Liver lipid extraction and LC–MS/MS analysis

A pre-cooled dichloromethane/methanol mixture (3:1, v/v) was used to extract lipids from 50 mg of liver tissue. Water was then added following the homogenization process, which would allow phase separation, and the mixture was centrifuged to extract the organic layer at the bottom. The lipid extract was then dried under vacuum and dissolved in isopropanol/acetonitrile/ water (2:1:1 v/v/v). The stability of the system and the reproducibility of the results were evaluated by preparing quality control (QC) samples obtained by mixing equal amounts of all the samples. To remove particulates before the liquid chromatography–tandem mass spectrometry (LC-MS/MS) analysis, the reconstituted solution was centrifuged at 13,000 × g at 4 °C for 10 min. A lipidomic analysis was conducted using a Q Exactive HF mass spectrometer (Thermo Fisher Scientific, United States of America) and operated in the positive and negative ion modes. LipidSearch software (version 4.1, Thermo Fisher Scientific) was used in the identification and quantification of lipids. The raw data of lipidomics profiling have also been deposited in the CNSA under the same accession number CNP0008322.

### 2.7 Analysis of metabolites

The application of PCA was performed on the stats package (v3.6.0) to evaluate the pattern of sample distribution. After that, t-tests were performed on the orthogonal partial least squares (OPLS) model, and the variable importance in projection (VIP) value of every metabolite was calculated, with an adjusted P-value <0.05, log2FC, and VIP >1, and visualized in volcano plots and heatmaps. This was performed using MetaboAnalyst [31] to conduct functional enrichment analysis and visualize the results of the significantly enriched metabolic pathways. The correlation of differential metabolites and biomarkers was performed using Spearman-rank correlation. The metabolite was identified as those with the significant correlations (P < 0.05 and 0.9) and the correlations of more than 0.9. The Wilcoxon rank-sum test was used to evaluate the difference in the abundance of these important metabolites between the MASH and control groups and visualized in box plots.

### 2.8 Hub genes expression validation in human MASH data

The validation dataset (GSE61260) was obtained at the National Center for Biotechnology Information (NCBI) Gene Expression Omnibus (GEO) database (Barrett et al., 2005) (GPL11532 platform, Affymetrix Human Gene 1.1 ST Array). The original GSE61260 dataset (Horváth et al., 2014; GEO accession: GSE61260) comprised 134 human liver samples from morbidly obese patients and healthy controls, with BMI ranging from 14.8 to 70.2 and ages from 10 to 85 years. The samples were grouped into control subjects (n = 38), healthy obese controls (n = 24), obese patients with non-alcoholic fatty liver (NAFL, n = 23), and obese patients with non-alcoholic steatohepatitis (NASH, n = 24). The processed expression matrix and corresponding platform annotation file were downloaded for probe-to-gene symbol conversion, with expression values averaged for multiple probes mapping to the same gene symbol. For the five candidate genes identified in our primary analysis, expression distributions in the validation cohort were visualized using box plots, and inter-group differences were assessed using the Wilcoxon rank-sum test. Genes with statistically significant differences (P < 0.05) and consistent expression patterns were considered validated. Diagnostic performance was evaluated by ROC curve analysis using the pROC package (v1.12.1) (Robin et al., 2011), and Spearman correlation analysis was performed among the validated biomarkers.

### 2.9 Mendelian randomization analysis

To examine the causal association between MASH hub genes and human MASH, we conducted a two-sample MR analysis. Based on stringent criteria (de Leeuw et al., 2015), instrumental variables (IVs) were selected from the eQTLGen Consortium meta-analysis of cis-eQTL data from 31,684 peripheral blood samples (Vosa et al., 2021) (false discovery rate <0.05 within ±1 Mb of each probe): 1) single-nucleotide polymorphism (SNP)-exposure association P-value <5 × 10^−8^; 2) elimination of linkage disequilibrium (r 2 = 0.1); 3) F = 10; and 4) outcome data on non-alcoholic fatty liver disease (NAFLD) were obtained from the genome-wide association study (GWAS) catalog (GCST90091033) (Ghodsian et al., 2021), consisting of 8,434 cases and 770,180 controls of European descent (N = 778,614). The TwoSampleMR package (v0.5.7) (Hemani et al., 2018) in R was used to perform MR analysis, and the main causal inference method is the inverse variance weighted (IVW) method (Pierce and Burgess, 2013). To achieve robustness, we conducted extensive sensitivity analyses; heterogeneity assessment was performed using MR-Egger (Bowden et al., 2015) regression and IVW (Pierce and Burgess, 2013); pleiotropy was tested using MR-Egger and MR-PRESSO regression (Verbanck et al., 2018), and leave-one-out analysis (Bowden et al., 2015) was performed to evaluate the impact of individual SNPs on the overall estimates.

### 2.10 Drug screening, molecular docking, and molecular dynamics simulations

Drug–gene interaction data for candidate genes were retrieved from DGIdb (Wagner et al., 2016) and GROEMINE (https://coremine.com/medical/) databases, and regulatory networks were constructed and visualized using Cytoscape v3.10.3 (Shannon et al., 2003). To elucidate the molecular mechanism of drug regulation, AutoDock Vina (Eberhardt et al., 2021) was used for docking simulations between target proteins (encoded by candidate genes) and candidate compounds. First, compound structures were obtained from PubChem (Kim et al., 2021) and protein structures from PDB (Karuppasamy et al., 2020), followed by dehydration, hydrogenation, and charge assignment using AutoDockTools and PyMOL (Seeliger and de Groot, 2010). Finally, AutoDock Vina software was used for molecular docking, and the results were visualized using PyMOL. Any binding free energy which was less than −1.2 kcal/mol (−5 kJ/mol) was taken to indicate strong binding affinity (Yin et al., 2020). Structural stability of the complex was also examined by 100 ns molecular dynamics (MD) simulations of the structure on GROMACS using the Linux platform (Van Der Spoel et al., 2005). The solvated sphere was used to generate the compound topology, and the system was neutralized with NaCl to 0.145 mol/L and solvated with Transferable Intermolecular Potential 3 Points (TIP3P) water model. Upon minimization and equilibration of the energy, production simulation was conducted at 310 K and 101 kPa. The stability of the system was determined by analyzing the root mean square deviation (RMSD), root mean square fluctuation (RMSF), number of hydrogen bonds, radius of gyration (Rg) and solvent-accessible surface area (SASA), and the analysis was presented using DuIvyTools.

### 2.11 *In vivo* experiments

Male C57BL/6J mice (Vital River Laboratory Animal Technology Co., Ltd., Beijing, China) were exposed to an AMLN diet over a 24-week period to develop MASH models, as earlier mentioned. After the induction phase, the mice were randomly divided into test groups where metformin (300 mg/kg/day; Sigma-Aldrich, Cat# D150959), apigenin (50 mg/kg/day; Shanghai Yuanye Bio-Technology Co., China, Cat# B20981), or both at the above doses were administered to the mice daily using intragastric injections for 4 weeks. At the end of the experiment, body and liver weights were determined. Blood samples were collected for serum biochemical analysis (ALT, AST, TG, and TCHO), and liver tissues were collected for histological study. For histopathological assessment, the liver tissues were briefly fixed, dehydrated, and paraffin-embedded for H&E and Sirius Red staining or snap-frozen in OCT compound for Oil Red O staining. Five-micrometer paraffin sections were dewaxed, stained with hematoxylin for 3 min and eosin for 15s, dehydrated, and mounted with neutral resin for H&E analysis. For Sirius Red staining, sections were incubated with Sirius Red A solution (ServiceBio, China, Cat# G1078-1) at 65 °C for 30 min, followed by modified Sirius Red B (ServiceBio, China, Cat# G1078-2) and C (ServiceBio, China, Cat# G1078-3) solutions for 2 min and 30 min, respectively, before dehydration and mounting. Eight-micrometer cryosections were stained with Oil Red O (ServiceBio, China, Cat# G1015) for 10 min, protected from light, differentiated in 60% isopropanol, counterstained with hematoxylin, and mounted with glycerol gelatin. All stained sections were examined and imaged under a microscope.

## 3 Results

### 3.1 Successful establishment of the MASH model

**FIGURE 1:**
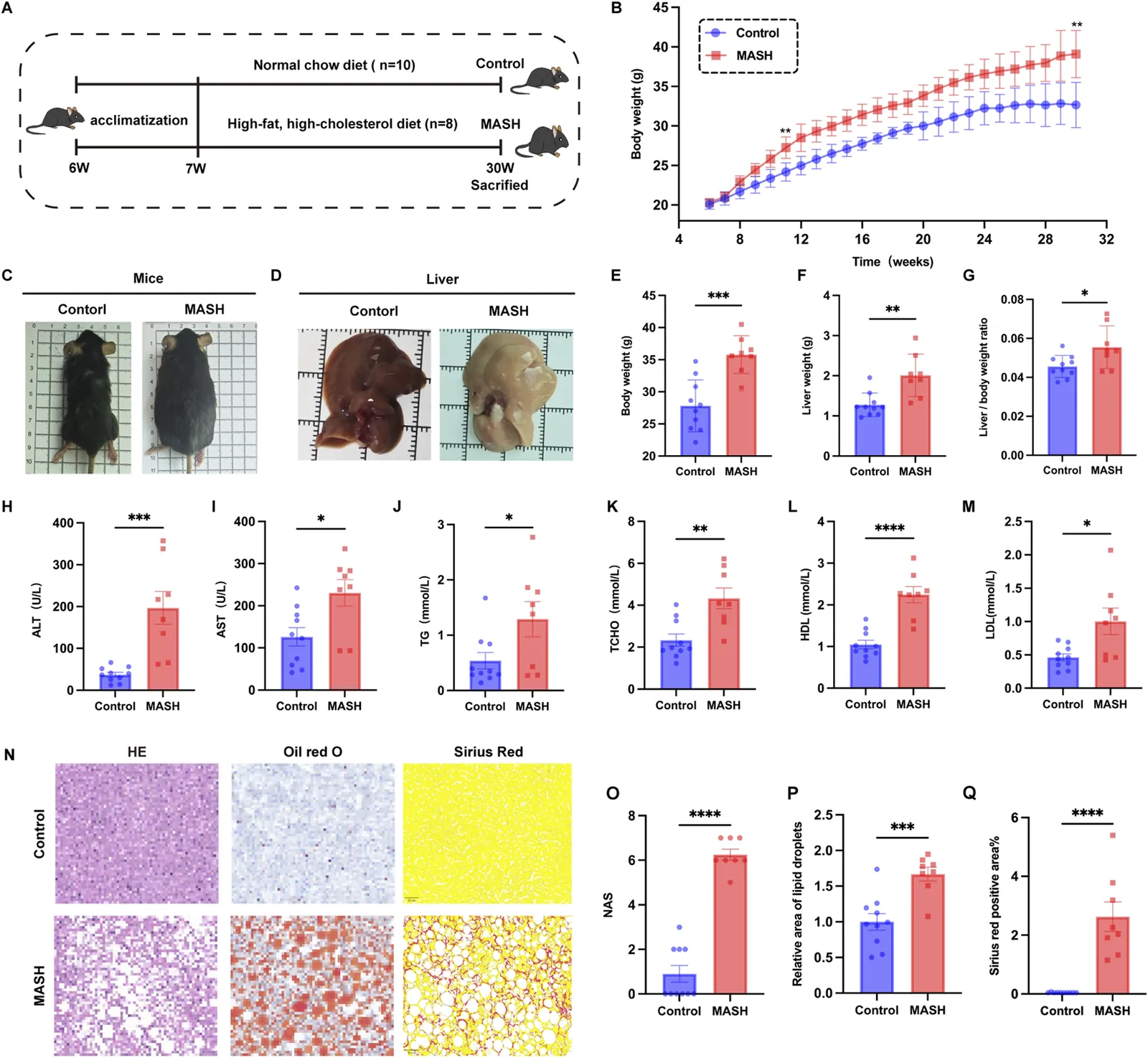
Induction and validation of MASH mice for sequencing. **(A)** Experimental protocols for induction of MASH mice. **(B)** Body weights of control mice and MASH mice over 30 weeks. Body weights were recorded every week. **(C,D)** Representative images of mice and liver for two groups. **(E–G)** Body weight, liver weight, and liver/body weight ratio of two groups. **(H–M)** Serum levels of ALT, AST, TG, TCHO, HDL, and LDL of two groups **(N)** Representative images of H&E, Oil red O, and Sirius red staining (scale bar, 50 µm). **(O–Q)** The analysis of NAS, relative area of lipid droplets, and Sirus red positive area of two groups (Control: n = 10, MASH: n = 8). Data are presented as mean ± SD and analyzed with a two-tailed unpaired Student’s t-test **(E–M,O–Q)** or two-way ANOVA **(B)**. *p < 0.05, **p < 0.01, ***p < 0.001, and ****p < 0.0001. MASH, metabolic dysfunction-associated steatohepatitis; ALT, alanine aminotransferase; AST, aspartate aminotransferase; TG, triglyceride. TCHO, total cholesterol; HDL, high-density lipoprotein; LDL, low-density lipoprotein; NAS, non-alcoholic steatohepatitis activity score.

The animal experiment using multi-omics analysis has a workflow, as schematically shown in Figure 1A. The MASH mouse model was effectively developed as depicted by a large body weight, liver weight, and serum of ALT, AST, TG, TCHO, HDL, and LDL relative to controls (Figures 1B–M). Histology also showed that in the MASH group, compared to controls, hepatic inflammation, steatosis, and collagen deposition were pronounced (Figures 1N–Q). These findings confirm that the model was well established in all the samples that were subjected to sequencing, which makes the latter multi-omics analysis reliable.

### 3.2 Clec12a, Cybb, Gpd2, Ifi211, and tifab were identified as hub genes

**FIGURE 2:**
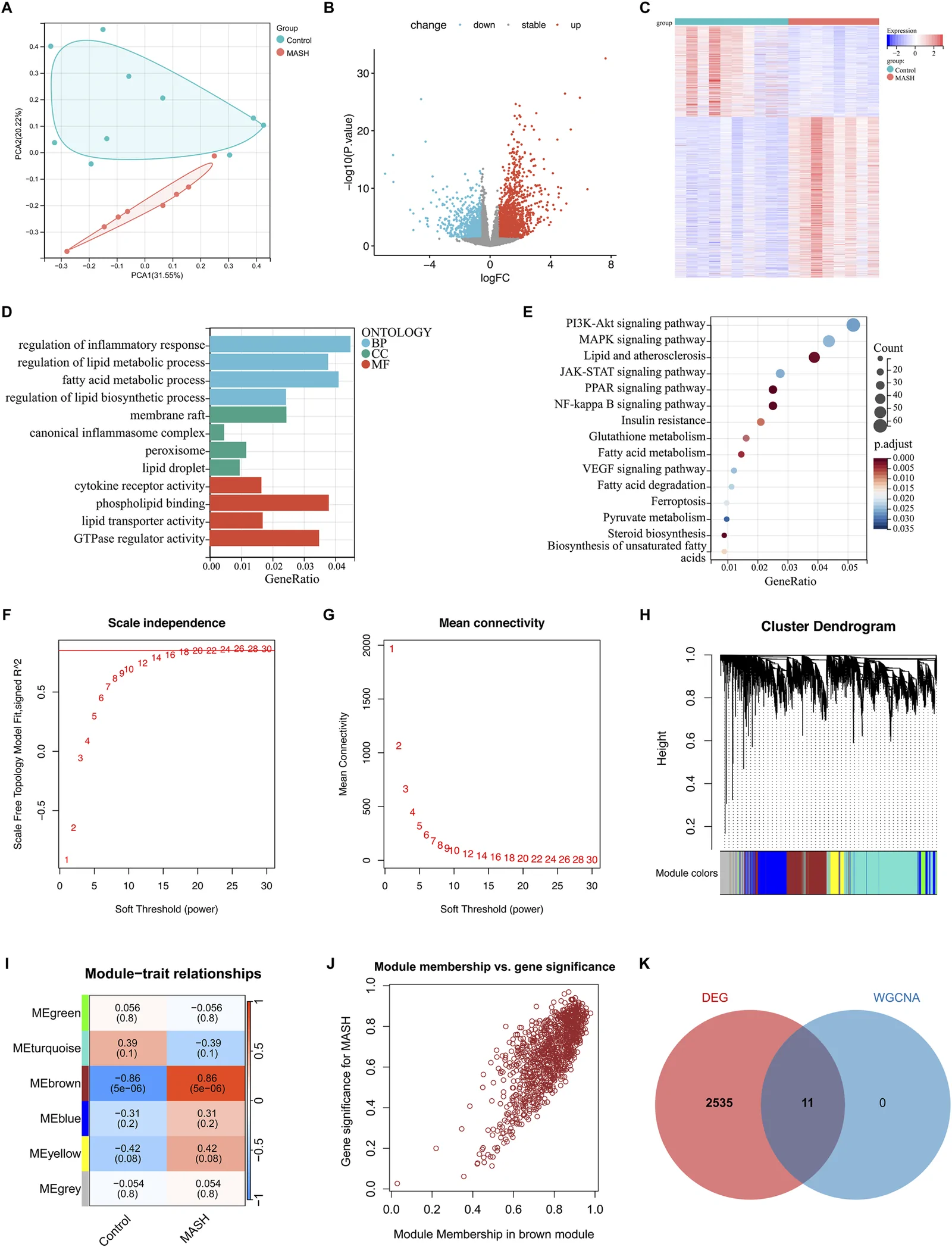
Transcriptomic analysis of DEGs, GO/KEGG, and WGCNA. **(A)** PCA distribution map of MASH and control groups in transcriptome data. **(B,C)** Volcano plot and heat map of DEGs between MASH and normal groups. **(D)** Bar plot of GO enrichment analysis of DEGs. **(E)** Bubble plot displaying KEGG pathway enrichment analysis of DEGs. **(F,G)** Scale-free fit index and average connectivity analysis with different soft thresholds of MASH groups. **(H)** MASH module cluster diagram. **(I)** The results of correlation analysis between WGCNA module and gene subgroup in MASH. **(J)** Correlation scatter plot of MM and GS values in key modules. **(K)** Venn diagram of DEGs and WGCNA. MASH, metabolic dysfunction-associated steatohepatitis; DEGs, differentially expressed genes; GO, Gene Ontology; KEGG, Kyoto Encyclopedia of Genes and Genomes; WGCNA, weighted gene co-expression network analysis; PCA, principal component analysis; MM, module membership; GS, gene significance.

The principal component analysis revealed a clear segregation between MASH and control groups (Figure 2A). Differential expression analysis identified 2,546 DEGs, comprising 1,627 upregulated and 919 downregulated genes (Supplementary Table S1), which were visualized via a volcano plot and a heatmap (Figures 2B,C). Functional enrichment analysis revealed 2,801 GO terms and 104 significantly enriched KEGG pathways (Supplementary Tables S2, S3). Notably, the most enriched GO categories encompassed the regulation of inflammatory response, regulation of lipid metabolic process, fatty acid metabolic process, and regulation of lipid biosynthetic process (BP); membrane raft, canonical inflammasome complex, peroxisome, and lipid droplet (CC); and cytokine receptor activity, phospholipid binding, lipid transporter activity, and GTPase regulator activity (MF) (Figure 2D). KEGG pathway enrichment revealed that the majority of significantly enriched pathways were concentrated in lipid metabolism and inflammation, including phosphatidylinositol 3-kinase-protein kinase (PI3K-Akt), AMP-activated protein kinase (MAPK), peroxisome proliferator-activated receptor (PPAR), nuclear factor-kappa B (NF-κB), fatty acid metabolism, and insulin resistance (Figure 2E). WGCNA was performed using a soft threshold power of 20 (Figures 2F,G) to cluster genes into six modules (Figure 2H). Among these, the brown module (1,021 genes) exhibited the strongest correlation with disease status (|r| = 0.86, P < 0.05) and was thus identified as the key module (Figure 2I; Supplementary Tables S4, S5). Further screening identified 11 hub genes with both module membership (MM) > 0.9 and gene significance (GS) > 0.9 within this module (Figure 2J; Supplementary Table S6). Remarkably, all 11 hub genes overlapped with the DEGs (Figure 2K; Supplementary Table S7), forming a robust candidate gene set for subsequent analysis.

**FIGURE 3:**
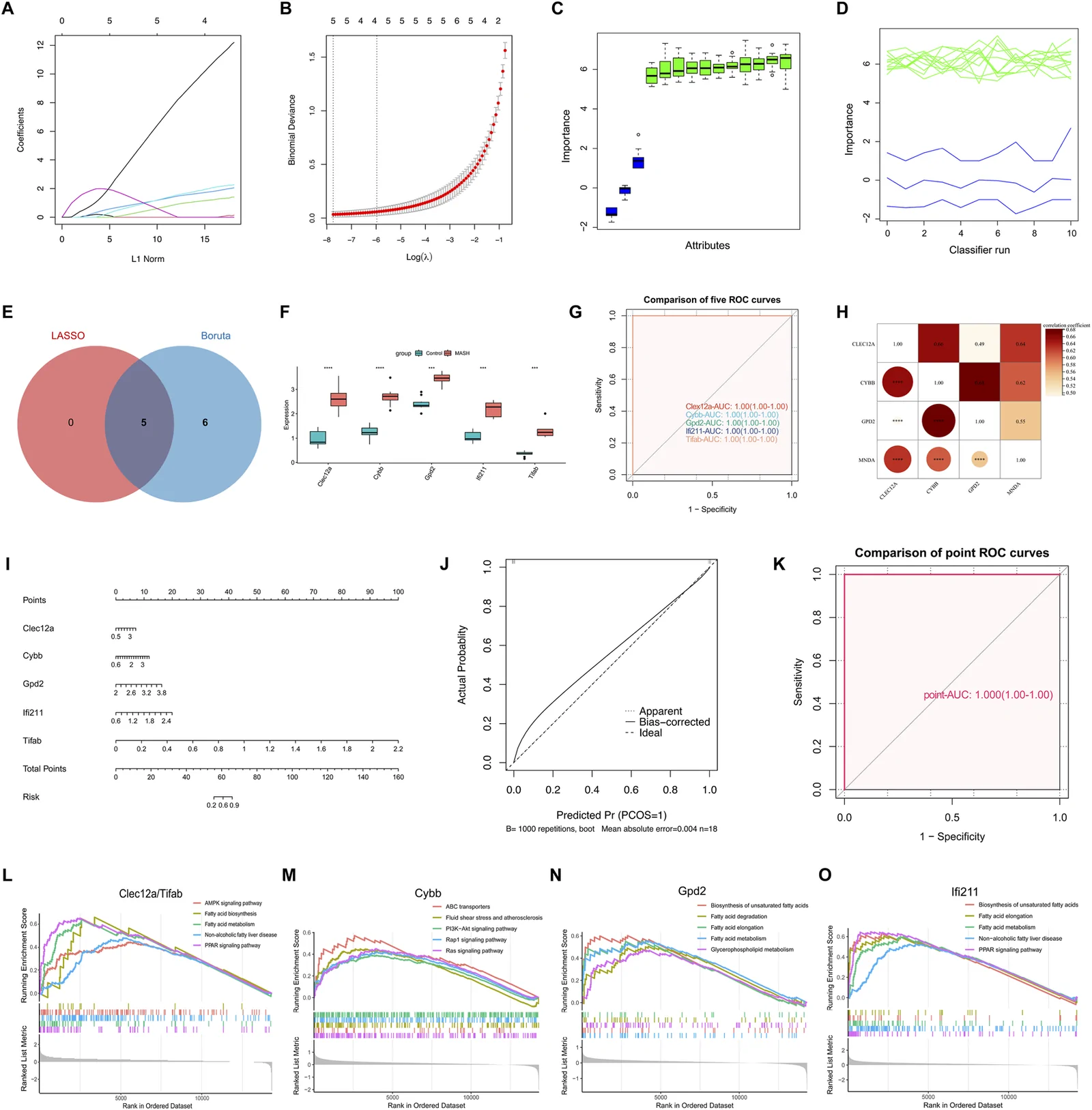
Hub genes identification, nomogram and GSEA analysis. **(A)** Distribution of the LASSO coefficients. **(B)** Likelihood bias of the LASSO coefficient distribution. **(C)** Boruta feature selection results showing final confirmed hub genes. Horizontal axis: candidate genes; vertical axis: importance values. Blue boxes represent reference metrics (minimum, median, and maximum). Green boxes indicate genes confirmed as significantly important (exceeding maximum reference metric). All displayed genes are confirmed (green). **(D)** Selection stability analysis across varying algorithmic parameters. Blue lines: reference metrics; green lines: confirmed genes. This panel demonstrates that hub gene importance remains consistently above selection thresholds regardless of parameter variation, validating the robustness of Panel C results. **(E)** Venn diagram of two types of machine learning. **(F)** Boxplot of the difference of hub genes between MASH and control groups. **(G)** ROC curve of hub genes. **(H)** Biomarker correlation heat map. **(I)** Nomogram of biomarker genes. **(J,K)** Calibration curve and ROC curve of the nomogram diagnostic model. AUC = 1.0 reflects the small training set size. **(L–O)** GSEA enrichment analysis across high- and low-expression groups of Clec12a/Tifab, Cybb, Gpd2, and Ifi211. ***p < 0.001 and ****p < 0.0001. GSEA, Gene Set Enrichment Analysis; LASSO, least absolute shrinkage and selection operator; MASH, metabolic dysfunction-associated steatohepatitis; ROC, receiver operating characteristic; Clec12a, C-type lectin domain family 12 member A; Tifab, TRAF-interacting protein with forkhead-associated domain B; Cybb, cytochrome b-245 beta chain; Gpd2, glycerol-3-phosphate dehydrogenase 2; Ifi211, interferon-activated gene 211.

To derive a clinically applicable biomarker signature, we applied LASSO and Boruta algorithms to the DEG matrix and identified five hub genes (Clec12a, Cybb, Gpd2, Ifi211, and Tifab) (Figures 3A–E; Supplementary Tables S8-S10). All five biomarkers were significantly upregulated in MASH (Figure 3F) and yielded an AUC of 1 (Figure 3G), with strong positive inter-correlations (Figure 3H). The AUC value of 1 is attributable to the small sample size of the transcriptome dataset, which results in the minimum value of the MASH group exceeding the maximum value of the control group. A five-gene nomogram was constructed (Figure 3I; Supplementary Table S11), with calibration plots closely following the diagonal (Figure 3J) and ROC AUC = 1 (Figure 3K), indicating excellent predictive accuracy. As all biomarkers are upregulated genes, the corresponding gene scores increase with gene expression, and a higher score indicates a greater risk of disease.

### 3.3 Enrichment of hub genes in lipid metabolic and inflammatory pathways

Subsequently, we performed GSEA for the identified hub genes. To highlight key findings, we focused on the most critical pathway categories relevant to MASH pathogenesis, including fatty acid metabolism, inflammatory signaling, and insulin resistance-related pathways (full lists provided in Supplementary Tables S12–S15).

Our analysis revealed that Clec12a and Tifab were significantly enriched in upregulated pathways, predominantly involving AMP-activated protein kinase (AMPK) signaling, fatty acid biosynthesis and metabolism, and the PPAR signaling pathway (Figure 3L). For Cybb, 32 significantly upregulated pathways were identified; notably, we highlight the PI3K–AKT and Ras signaling pathways, which share strong connections with lipid metabolism and inflammation (Figure 3M). Regarding Gpd2, significant enrichment was observed in lipid-related routes, specifically unsaturated fatty acid biosynthesis, fatty acid degradation, and glycerophospholipid metabolism (Figure 3N). Furthermore, Ifi211 exhibited broad pathway activation, with lipid metabolism-related terms—such as those associated with NAFLD and the PPAR signaling pathway—being highly upregulated (Figure 3O). Collectively, these results suggest that these hub genes may play pivotal roles in MASH progression by coordinately regulating lipid metabolic reprogramming and inflammatory responses.

Immune infiltration analysis was performed but showed no significant association with subsequent mechanistic findings. These data are provided in Supplementary Figures S1A,B and Supplementary Tables S16,S17 for completeness.

### 3.4 Correlation between hub genes and specific lipid metabolites

**FIGURE 4:**
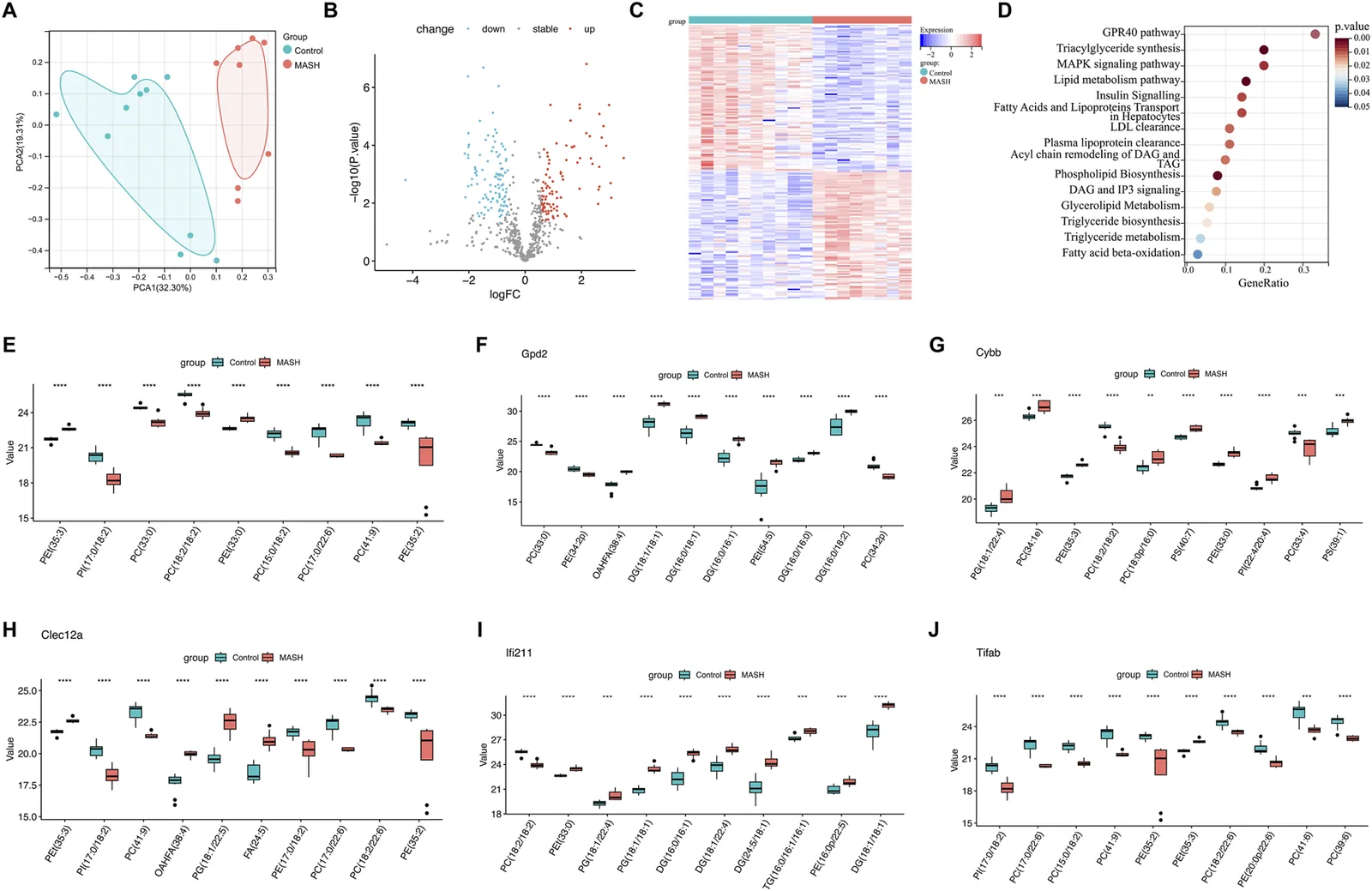
Results of lipid metabolomic analysis. **(A)** PCA distribution map of MASH and control groups in lipid metabolomics data. **(B,C)** Volcano plot and heat map of differential metabolites between MASH and control groups. **(D)** KEGG pathway enrichment of differential metabolites. **(E)** Box plot of differences in key metabolites. **(F–J)** Box plot of differences in key metabolites closely related to Gpd2, Cybb, Clec12a, Ifi211, and Tifab. **p < 0.01, ***p < 0.001, and ****p < 0.0001. PCA, principal component analysis; MASH, metabolic dysfunction-associated steatohepatitis; KEGG, Kyoto Encyclopedia of Genes and Genomes; Gpd2, glycerol-3-phosphate dehydrogenase 2; Cybb, cytochrome b-245 beta chain; Clec12a, C-type lectin domain family 12 member A; Ifi211, interferon-activated gene 211; Tifab, TRAF-interacting protein with forkhead-associated domain B.

Since our biomarkers are closely associated with lipid metabolism, we performed lipidomic analysis and correlated the hub genes with lipid metabolites. PCA of the metabolomic dataset showed clear separation between MASH and control samples (Figure 4A). Differential analysis identified 200 metabolites; among these, 93 were upregulated, and 107 were downregulated (Supplementary Table S18). Volcano and heat maps are shown in Figures 4B,C, respectively.

For KEGG enrichment, metabolite IDs were converted to HMDB IDs (Supplementary Table S19) before enrichment analysis. The most enriched pathways included G protein-coupled receptor 40 (GRP40) signaling, triacylglyceride synthesis and metabolism, MAPK signaling, acyl-chain remodeling of diacylglycerol (DG) and TG, DG and inositol 1,4,5-trisphosphate (IP3) signaling, fatty acid β-oxidation, and fatty acid and lipoprotein transport in hepatocytes (Figure 4D; Supplementary Table S20). The three major metabolites in these pathways were DG (16:0/16:0), phosphatidylinositol (PI) (16:0/16:1 (9Z)), and TG (16:0_32:1), with TG (16:0_32:1) corresponding to TG (16:0/16:0/16:1) and PI (16:0/16:1 (9Z) to PI (16:0/16:1).

Correlation analysis between differential metabolites and biomarkers identified nine key metabolites (|r| > 0.9 and significantly correlated with at least one biomarker), visualized as differential box plots (Figure 4E). Except for PEt (35:3) and PEt (33:0), all were significantly downregulated (Supplementary Table S21). Gpd2 negatively correlated with phosphatidylcholine (PC) (33:0), phosphatidyl ethanolamine (PE) (34:2p), and PC(34:2p) and positively with O-acyl-ω-hydroxy fatty acid (OAHFA) (38:4), DG (18:1/18:1), DG (16:0/18:1), DG (16:0/16:1), PEt (54:5), DG (16:0/16:0), and DG (16:0/18:2) (Figure 4F). Cybb negatively correlated with PC(18:2/18:2) and PC(33:4) and positively with phosphatidylglycerol (PG) (18:1/22:4), PC (34:1e), PEt (35:3), PC (18:0p/16:0), PS (40:7), PEt (33:0), PI (22:4/20:4), and phosphatidylserine (PS) (39:1) (Figure 4G). Clec12a negatively correlated with PI (17:0/18:2), PC( 41:9), PE (17:0/18:2), PC (17:0/22:6), PC (18:2/22:6), and PE (35:2) and positively with PEt (35:3), OAHFA (38:4), PG (18:1/22:5), and FA (24:5) (Figure 4H). Ifi211 negatively correlated with PC(18:2/18:2) and positively with PEt (33:0), PG (18:1/22:4), PG (18:1/18:1), DG (16:0/16:1), DG (18:1/22:4), DG (24:5/18:1), TG (16:0/16:1/16:1), PE (16:0p/22:5), and DG (18:1/18:1) (Figure 4I). Tifab negatively correlated with PI (17:0/18:2), PC (17:0/22:6), PC (15:0/18:2), PC (41:9), PE (35:2), PC (18:2/22:6), PE (20:0p/22:6), PC (41:6), and PC (39:6) and positively with PEt (35:3) (Figure 4J).

### 3.5 Upregulation of hub genes in liver samples from MASH patients

**FIGURE 5:**
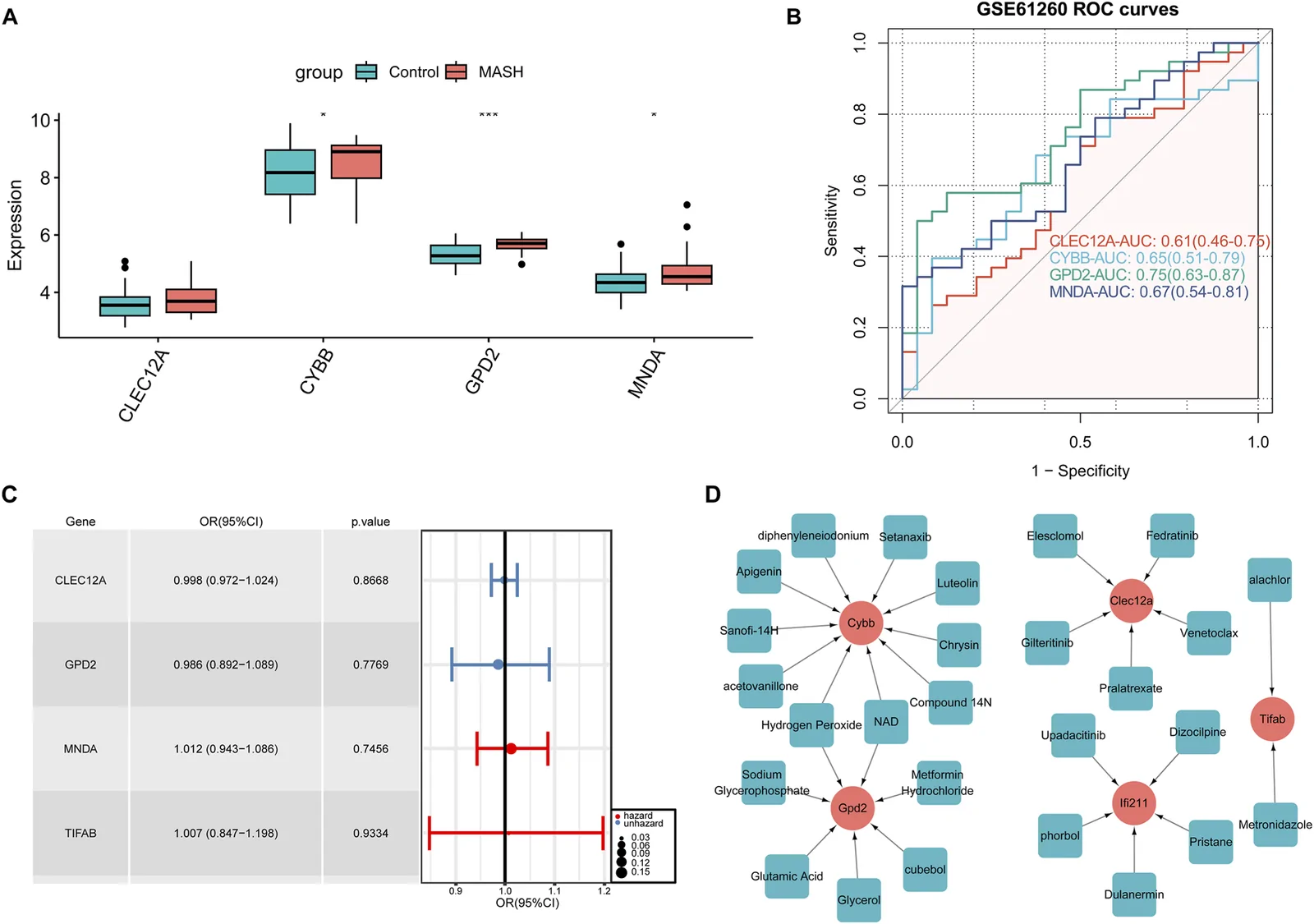
Validation of hub genes in human liver datasets, MR analysis, and drug prediction. **(A)** Boxplot of the difference in hub genes between MASH and control groups in the human liver dataset. **(B)** ROC curve of hub genes in the validation dataset. **(C)** Forest plot of Mendelian randomization (MR) results for hub genes and MAFLD. **(D)** Small-molecule drug regulation map of the hub genes. *p < 0.05 and ***p < 0.001. MR, Mendelian randomization; MASH, metabolic dysfunction-associated steatohepatitis; ROC, receiver operating characteristic; MAFLD, metabolic dysfunction-associated fatty liver disease. CLEC12A, C-type lectin domain family 12 member A; GPD2, glycerol-3-phosphate dehydrogenase 2; MNDA, myeloid nuclear differentiation antigen; TIFAB, TRAF-interacting protein with forkhead-associated domain B.

To validate the expression of the selected biomarkers in human MASH, we analyzed the public transcriptome dataset GSE61260 from the GEO database (Supplementary Tables S22, S23). After orthologous conversion (Ifi211→MNDA) and excluding the absent Tifab gene, four hub genes were tested. All genes were upregulated in the MASH group, with significant differences observed for three of them, except for Clec12a (P < 0.05, Figure 5A; Supplementary Table S24). ROC analyses yielded AUC values >0.6 (Figure 5B), and pairwise correlations remained significantly positive (Supplementary Figure S1C), confirming the cross-species conservation and diagnostic utility of these hub genes in human MASH. These findings, replicated in human liver samples, strengthen the credibility of the identified biomarkers as potential therapeutic targets for MASH and provide a solid foundation for the subsequent Mendelian randomization analysis.

We further analyzed the expression levels of Cybb and Gpd2 across control, obese, MAFL, and MASH groups. As shown in Supplementary Figure S2, Cybb showed no differential expression between healthy controls and obese individuals. However, Cybb was significantly elevated in both MAFL and MASH compared with controls, with no further increase between MAFL and MASH (P < 0.05). In contrast, Gpd2 showed no significant differences among healthy controls, obese individuals, and MAFL patients but was significantly elevated in MASH compared with all other groups (P < 0.05).

### 3.6 Absence of causal association between hub genes and MASH in Mendelian randomization analysis

To determine whether the five candidate genes exert a causal, genetically driven influence on MASH, we performed two-sample MR. Orthologous conversion of the five murine biomarkers (Clec12a, Cybb, Gpd2, Ifi211, and Tifab) yielded their human equivalents: Clec12a, Cybb, Gpd2, MNDA, and Tifab. After strict quality control, valid IVs were available for four of the five loci, except Cybb.

Inverse-variance-weighted results revealed no significant causal effect of any examined gene on MAFLD risk (Figure 5C; Supplementary Table S25). Forest and scatter plots provided in Supplementary Figures S3A-H illustrate the causal estimates for each gene. Sensitivity analysis of each gene showed neither directional pleiotropy (MR Egger and MR-PRESSO, P > 0.05, Supplementary Table S26) nor inter-SNP heterogeneity (MR Egger and IVW, Q_pval >0.05 Supplementary Figures S3I-L; Supplementary Table S27), and leave-one-out analysis showed that removal of any single instrumental variable did not materially alter the pooled estimate for any gene (Supplementary Figures S3M-P). Collectively, these diagnostics support robust and unbiased MR estimates that do not indicate a causal relationship between the investigated biomarkers and MAFLD/MASH development.

### 3.7 Molecular docking of metformin and apigenin to Gpd2 and Cybb

**FIGURE 6:**
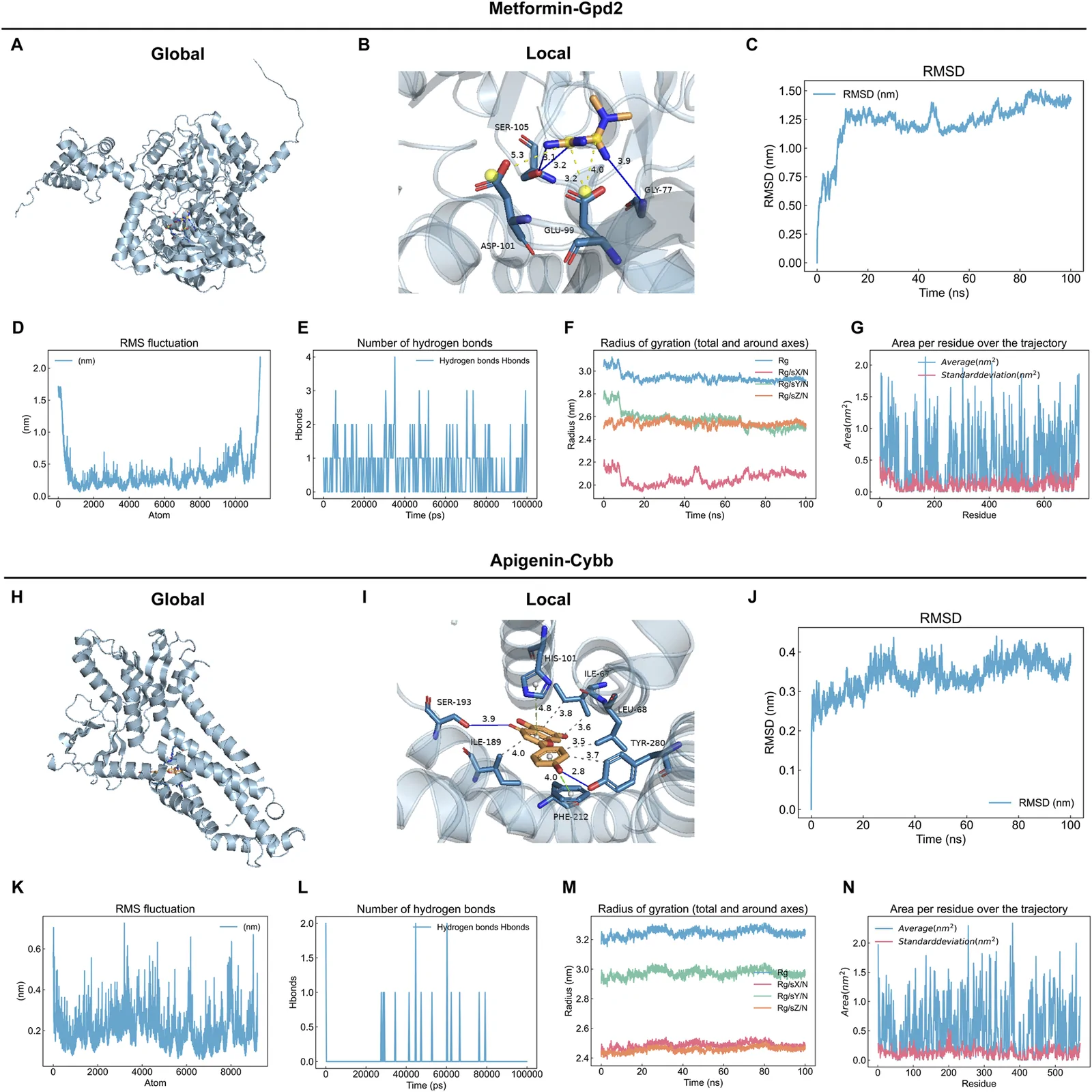
Molecular docking and molecular dynamics simulation of metformin–Gpd2 and apigenin–Cybb. **(A,B)** Global and local molecular docking of metformin and Gpd2. **(C–G)** The RMSD, RMSF, hydrogen bond number, Rg value, and surface area of metformin and Gpd2. **(H,I)** Global and local molecular docking of apigenin and Cybb. **(J–N)** The RMSD, RMSF, hydrogen bond number, Rg value, and surface area of apigenin and Cybb. Gpd2, glycerol-3-phosphate dehydrogenase 2; Cybb, cytochrome b-245 beta chain; RMSD, root mean square deviation; RMSF, root mean square fluctuation; Rg, radius of gyration.

Drug–gene interactions retrieved from DGIab (Supplementary Table S28) and GROEMINE (Supplementary Table S29) databases were integrated to construct a regulatory network (Figure 5D; Supplementary Table S30). One representative ligand per biomarker was docked to its target protein: metformin–Gpd2 (−6 kcal/mol; Figures 6A,B), apigenin–Cybb (−9.1 kcal/mol; Figures 6H,I), venetoclax–Clec12a (−8.7 kcal/mol; Supplementary Figures S4A,B), upadacitinib–Ifi211 (−7.6 kcal/mol; Supplementary Figures S4C,D), and metronidazole–Tifab (−4.4 kcal/mol; Supplementary Figures S4E,F). Binding energies for all drug–target complexes are listed in Supplementary Table S31. The two most favorable complexes (metformin–Gpd2 and apigenin–Cybb) were subjected to 100-ns MD simulation, and they both exhibited low fluctuations in RMSD, RMSF, hydrogen-bond number, radius of gyration, and solvent-accessible surface area (metformin–Gpd2 Figures 6C–G, and apigenin–Cybb Figures 6J–N), confirming stable binding.

### 3.8 Metformin–apigenin combination therapy outperforms monotherapy *in vivo*

**FIGURE 7:**
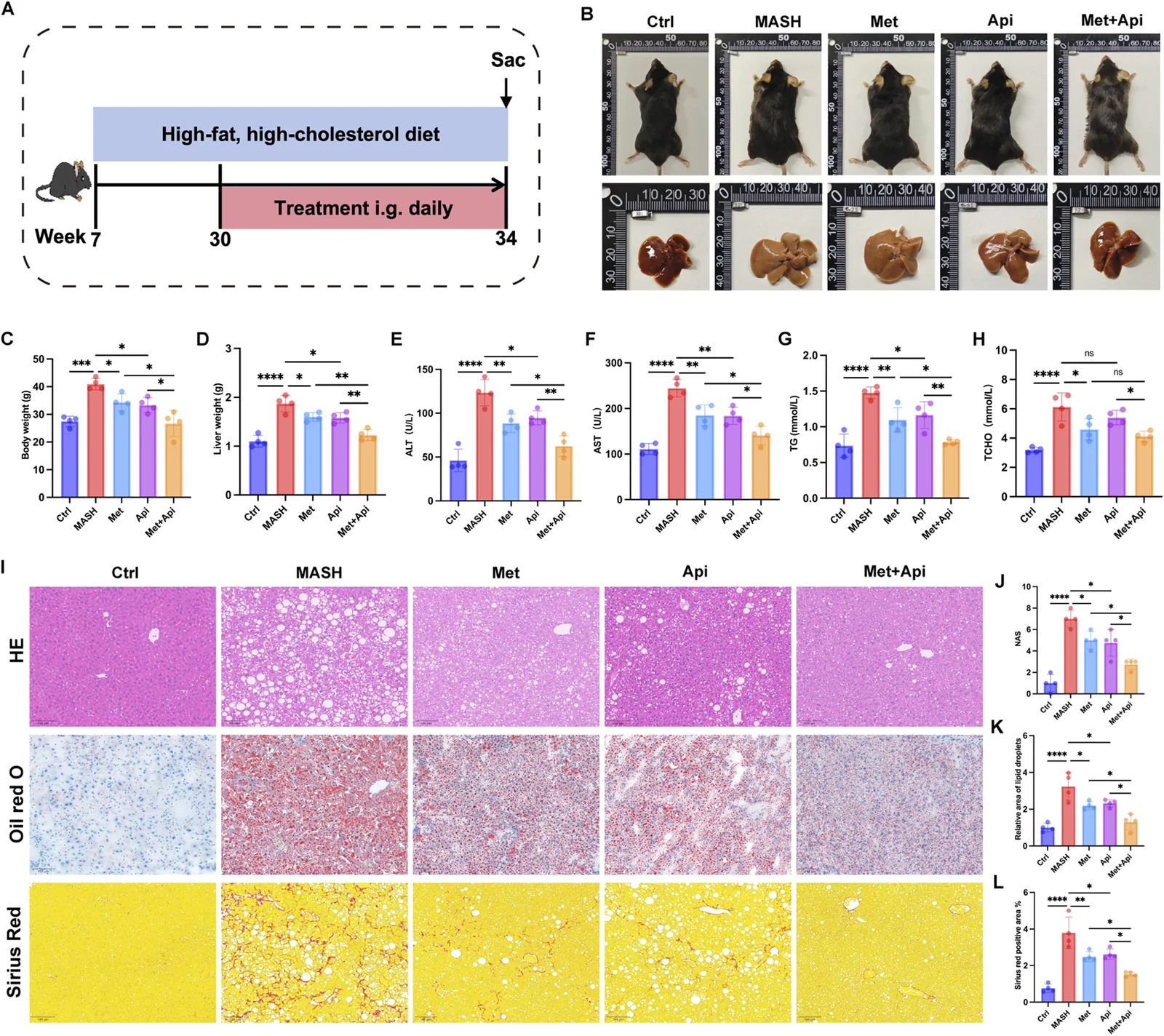
*In vivo* therapeutic efficacy. **(A)** The scheme of treatment with MASH mice. **(B–D)** The body weight and liver weight of MASH mice. **(E–H)** The serum levels of ALT, AST, TG, and TCHO. **(I)** Representative images of H&E, Oil red O, and Sirius red staining (scale bar, 100 µm). **(J–L)** The analysis of NAS, relative area of lipid droplets, and Sirus red positive area of each group (n = 4). Data are presented as mean ± SD and analyzed with one-way ANOVA. *p < 0.05, **p < 0.01, ***p < 0.001, and ****p < 0.0001, n. s.: not significant. Ctrl, control; MASH, metabolic dysfunction-associated steatohepatitis; Met, metformin; Api, apigenin; Met + Api, metformin + apigenin. ALT, alanine aminotransferase; AST, aspartate aminotransferase; TG, triglyceride. TCHO, total cholesterol; NAS, non-alcoholic steatohepatitis activity score.

The HFD-induced liver MASH model was used to evaluate its efficacy in the treatment of MASH. After the establishment of the liver MASH model, the mice were treated for 4 weeks (Figure 7A). The results of in vivo experiments showed that metformin and apigenin could significantly reduce the body weight and liver weight of MASH mice (Figures 7B–D) and reduce the levels of ALT (Figure 7E), AST (Figure 7F), and TG (Figure 7G) in serum. TCHO levels were significantly reduced in the Met group compared with the MASH group, whereas no statistically significant difference was observed between the Api and MASH groups. Compared with the Met group, the Met+Api group showed no statistically significant difference in TCHO levels; however, compared with the Api group, the Met+Api group significantly reduced TCHO levels (Figure 7H). Histopathological examination showed that liver inflammation (Figures 7I,J), steatosis (Figures 7I,K), and fibrosis (Figures 7I,L) were significantly improved after treatment. It is worth noting that compared with monotherapy, combination therapy showed a significantly enhanced therapeutic effect.

## 4 Discussion

In the present study, we employed an integrated multi-omics approach combining transcriptomic and lipidomic profiling to identify a five-gene signature (Gpd2, Cybb, Clec12a, Ifi211, and Tifab) intimately associated with lipid metabolism and inflammatory processes in MASH. Notably, Mendelian randomization indicated no simple genetic causality with MAFLD, suggesting that their involvement in MASH entails complex regulatory mechanisms beyond simple genetic determinism. Of significant translational importance, molecular docking analyses identified metformin as a high-affinity ligand of Gpd2 and apigenin as a predicted binder of Cybb, marking the first computational demonstration of apigenin’s potential interaction with this oxidase. *In vivo* experiments confirmed that these agents significantly ameliorate MASH pathology, with combination therapy demonstrating superior efficacy compared to monotherapy. To the best of our knowledge, this study is the first attempt to suggest a metformin–apigenin combination treatment regimen of MASH.

These five hub genes were originally identified from AMLN diet-induced MASH mouse model sequencing data and subsequently validated in human liver tissue samples. The AMLN diet, rich in trans-fat, cholesterol, and fructose, mimics the Western dietary pattern and induces progressive MASH with obesity, insulin resistance, and fibrosis, thereby closely recapitulating human disease driven by processed food consumption (Clapper et al., 2013). However, we observed a marked performance discrepancy between the training set (AUC = 1.0) and the human validation set (AUC ≈0.6–0.7). This disparity primarily reflects three interconnected factors. First, small sample sizes induce “AUC optimism,” where complete group separability in the training set inflates apparent performance beyond true cross-population applicability (Efthimiou et al., 2024; Kim et al., 2025). Second, species-specific biological heterogeneity constrains cross-species generalization; as previously reported, no single murine model fully recapitulates human MASH transcriptomic features, consistent with observed declines in cross-species predictive performance (Ning et al., 2025). Third, training genes were selected through differential expression screening (|log2FC| > 0.585, adjusted P < 0.05) without pre-screening in human datasets, where species-specific transcriptional regulation and batch effects may reshape expression distributions. Notably, validation AUC maintained at 0.6–0.7 indicates retained discriminatory capacity beyond random chance, supporting potential biological significance. This translational gap underscores the necessity of integrating multi-model consensus strategies and domain adaptation methods to bridge preclinical findings with human disease while also highlighting the value of our validated five-gene signature in MASH. Furthermore, our MR analysis provided additional insights into the complex causal relationships of these genes. With the exception of Cybb, for which no valid instrumental variables could be constructed due to the lack of genome-wide significant cis-eQTLs, none of the other hub genes exhibited a significant causal relationship with MASH. These null MR results may be attributed to several mechanisms. Specifically, blood-derived expression quantitative trait loci may fail to capture liver-specific regulatory patterns of gene expression. Additionally, there may be a lack of sufficiently strong genetic instruments for reliable inference. Moreover, these genes may represent downstream consequences of MASH pathogenesis rather than upstream causal drivers. These limitations underscore that MR serves as a complementary tool to transcriptomic and experimental evidence, rather than a definitive arbiter of causality. This finding further corroborates that the involvement of these multi-omics signature genes in MASH entails complex regulatory mechanisms beyond simple genetic determinism. Notably, validation AUC maintained at 0.6–0.7 indicates retained discriminatory capacity beyond random chance, supporting potential biological significance. This translational gap underscores the necessity of integrating multi-model consensus strategies and domain adaptation methods to bridge preclinical findings with human disease while also highlighting the value of our validated five-gene signature in MASH.

One of the crucial enzymes anchored to the outer surface of the inner mitochondrial membrane is mitochondrial glycerol-3-phosphate dehydrogenase 2 (Gpd2, or mGPD) that catalyzes the oxidation of glycerol-3-phosphate (G3P) to dihydroxyacetone phosphate (DHAP) and feeds electrons to the electron transport chain (Yeh et al., 2008). This response places Gpd2 at an essential crossroads of metabolism between glycolysis, lipid metabolism, and mitochondrial oxidative phosphorylation (Yeh et al., 2008). Our transcriptomic study indicated that Gpd2 is significantly upregulated in both MASH mouse liver and human patient samples, indicating its cross-species relevance. This observation aligns with findings by Silva-Gaona et al. (2022), who demonstrated through proteomic analysis of mouse livers fed a high-fructose diet that Gpd2 protein expression increased 16-fold. High fructose intake is a well-established driver of MASH pathogenesis, promoting MASLD onset and progression and inducing MASH-like inflammation (Li et al., 2024). Additionally, Zu et al. (2026) reported that GPD2-knockout mice exhibited reduced adiposity and lower hepatic lipid accumulation under high-fat diet feeding, suggesting that Gpd2 upregulation contributes to steatosis progression. Nevertheless, the involvement of Gpd2 in MASLD remains debated. Zheng et al. (2019) showed that Gpd2 levels and activity were downregulated in fatty livers of patients and murine models and that Gpd2 deficiency counterintuitively worsened hepatic steatosis and inflammation. Our validation in human liver tissue samples provides further insights into these discrepancies. Across control, obese, MAFL, and MASH groups, we found no significant differences in Gpd2 levels between healthy controls and the obese or MAFL groups; however, Gpd2 was significantly elevated in the NASH group compared to that in all other groups. This result suggests that Gpd2 upregulation may occur predominantly during the critical transition from simple steatosis to MASH, thereby explaining the divergent expression patterns observed in previous studies. Such differences likely reflect disease stage-specific expression patterns: Gpd2 may be suppressed in early steatosis as a compensatory mechanism but increased during the transition to steatohepatitis when mitochondrial dysfunction and inflammation become more pronounced. The results of our lipidomic correlation analysis indicated that Gpd2 expression has a positive correlation with DG–a thoroughly established lipotoxic species that promotes protein kinase C, insulin signaling, oxidative stress, and chronic inflammation and, thus, increases MASH progression (Byrne et al., 2025). Conversely, the expression of Gpd2 is negatively correlated with PC and PE, whose reduced levels are linked to the impairment of membrane integrity of hepatocytes, lobular inflammation, hepatocellular ballooning, and fibrosis (Li et al., 2006; Ogawa et al., 2020; Anari and Montgomery, 2023; Kim et al., 2023). The pattern of this correlation is that high Gpd2 in MASH could be correlated with lipotoxic lipid accumulation, but the causal relationship between the two needs further functional confirmation.

The catalytic subunit gp91phox of NADPH oxidase 2 (NOX2), which is the main source of reactive oxygen species (ROS) that drives inflammation and oxidative stress during MASH pathogenesis, is encoded by Cybb (cytochrome b-245 beta chain) (Day and James, 1998; Takaki et al., 2013; Cammisotto et al., 2025). It is important to note that it is the most researched gene in MASH in our five-gene signature. We have shown that Cybb was highly upregulated in both liver tissues of MASH mouse models and human patients, which is in line with many prior studies that reported an upregulation in NOX2 expression and activity of preclinical and clinical cohorts (Day and James, 1998; Takaki et al., 2013; Del Ben et al., 2014; Dattaroy et al., 2016; Loffredo et al., 2017; Jiang et al., 2020; Grossini et al., 2021). Clinical studies also indicate that the levels of serum soluble NOX2-derived peptide (sNOX2-dp) are in linear correlation with histological severity parameters such as steatosis, inflammation, ballooning degeneration, fibrosis, and NAFLD activity score (Loffredo et al., 2019). More importantly, Cybb/NOX2 is a key mediator of malignant change in the course of MASH progression to HCC (Wang et al., 2022). In line with these pathogenic activities, interventional studies on NOX2-deficient or p41phox-knockout models indicate that attenuation of NOX2 signals prevents high-fat-diet-induced metabolic imbalance, which is characterized by reduced steatosis, enhanced insulin sensitivity, normal mitochondrial function, and reduced progression of MASH with reduced inflammation and hepatic stellate cell stimulation (Garcia-Ruiz et al., 2016; Kim et al., 2017; Albadrani et al., 2019). In addition to determined functions, our lipidomic findings showed new connections between Cybb expression and lipid homeostasis dysregulation. Positively correlated relationships were found between Cybb and pro-inflammatory PG, PI, PS products, and anti-inflammatory PC products were negatively correlated. These results provide a mechanistic connection between the activation of NOX2 and pathological lipid remodeling in MASH, which strengthens Cybb as a target of therapeutic intervention by drug.

C-type lectin domain family 12 member A (Clec12a) (also called CLL-1) is an anti-adhesive C-type lectin receptor, which is expressed mainly on myeloid cells and is involved in the inhibition of SYK-mediated activation signals through ITIM-recruited SHP-1/SHP-2 phosphatases (Drouin et al., 2020; Man and Kanneganti, 2024). Traditionally, Clec12a is an inflammation brake that detects damage-related molecular patterns, including uric acid crystals (Neumann et al., 2014; Bordon, 2024; Chiaro et al., 2025). The recent research has shown that in chronic viral infections, Clec12a can contribute to the amplification of type I interferon (IFN-I) signaling through the Src–TBK1–IRF3 pathway, which aggravates chronic immunopathology in which inhibition would be effective (Wilson et al., 2013; Li K. et al., 2019). Our study will be the first illustration of hepatic Clec12a upregulation in MASH mice and its specific lipidomic correlations: positive correlations with pro-inflammatory ether-phospholipids and negative correlations with PC and PE species. These findings suggest that therapeutic inhibition of Clec12a might paradoxically benefit chronic MASH by dampening sustained IFN-I responses and restoring metabolic homeostasis, representing a novel immunometabolic therapeutic angle that is intriguing but requires further experimental validation.

Interferon-inducible protein 211 (Ifi211, in humans known as myeloid nuclear differentiation antigen, MNDA) belongs to the interferon-inducible protein family (Bottardi et al., 2024). Although direct evidence linking Ifi211 to MASH is currently lacking, its homolog IFI16 has been firmly established as a driver of MASLD progression via the IFI16–PYCARD–CASP1 axis, promoting macrophage inflammation and mitochondrial dysfunction and serving as a potential tissue and peripheral blood biomarker (Kim et al., 2025). Our study reveals, for the first time, robust upregulation of Ifi211 in both mouse and human NASH livers. Integrative lipidomics further demonstrated strong positive correlations with lipotoxic species (DG, TG, and PE) and an inverse correlation with PC, suggesting its involvement in lipid remodeling. As a canonical type I interferon-stimulated gene, Ifi211 expression is transcriptionally regulated by IFN-I signaling (Ji et al., 2023). The concurrent upregulation of Ifi211 with Clec12a in our results suggests persistent IFN-I signaling activation during MASH pathogenesis, a relatively underexplored and underappreciated aspect of MASH immunopathology that warrants further investigation.

It should be specifically noted that in the human cohort, the expression difference in Clec12a did not reach statistical significance and that Tifab was absent from this dataset. We believe that this phenomenon may be attributed to the following factors. First, dataset selection and technical heterogeneity: different platforms and processing protocols across datasets yield variable detection sensitivity and gene coverage. Second, species heterogeneity and human disease complexity: standardized mouse models amplify consistent gene signals, whereas human MASH exhibits substantial inter-individual variation that may dilute biomarker signals detectable in bulk transcriptomics. Therefore, although animal models provide important mechanistic clues, their translational application still requires validation in larger-scale, more representative clinical cohorts.

Tumor necrosis factor (TNF) receptor-associated factor (TRAF)-interacting protein with forkhead-associated domain B (Tifab) functions as an inhibitor of NF-κB signaling and has been studied primarily in the context of hematopoietic malignancies (Varney et al., 2015; Niederkorn et al., 2020; Zhao et al., 2021; Nakamura et al., 2024). Our study extends the investigation of Tifab to metabolic liver disease for the first time, revealing significant upregulation in MASH mouse liver tissues. Integrative lipidomics identified positive correlations with ether-phosphatidylethanolamine species and negative correlations with PC and PE species, suggesting its involvement in pathological lipid remodeling. Similar to its role in acute myeloid leukemia, where Tifab-mediated metabolic reprogramming provides survival to diseased cells and disease progression (Wang Y. et al., 2025), Tifab-based metabolic remodeling in MASH may also play a comparable role in promoting metabolic disturbances essential to disease pathogenesis, highlighting the translational utility of targeting metabolic adaptors in chronic inflammatory liver disease.

There are important therapeutic implications of our findings. Molecular docking revealed that metformin exhibited high predicted binding affinity to Gpd2, while apigenin showed potential binding interaction with Cybb. *In vivo* experiments confirmed that these agents individually ameliorate MASH pathology. Metformin, a biguanide antihyperglycemic agent and first-line therapy for T2DM (Bailey et al., 2026), has garnered significant interest for its potential therapeutic role in MASLD beyond its conventional antidiabetic use (Perazza et al., 2024). However, clinical trials in MASH have yielded inconsistent results: while some studies suggest modest improvements in liver enzymes and hepatic fat content, others demonstrate limited impact on histological features (Li et al., 2013; Perazza et al., 2024). These variable clinical responses may reflect an incomplete understanding of the molecular mechanisms underlying metformin’s direct actions on hepatic metabolism. Traditionally, metformin has been thought to act through the inhibition of mitochondrial respiratory chain complex I and subsequent AMPK activation (He et al., 2026), but recent studies suggest that metformin may primarily inhibit complex IV, leading to electron backflow and indirect suppression of Gpd2 activity (LaMoia et al., 2022). Although some studies have proposed that metformin may function through direct targeting of Gpd2 (Madiraju et al., 2014; Thakur et al., 2018), Gpd2 is a controversial *bona fide* direct target (Alshawi and Agius, 2019; MacDonald et al., 2021). Our molecular docking analysis provides novel structural insights supporting Gpd2 as a candidate target for metformin. This observation is particularly relevant because autophagy dysfunction drives lipid accumulation and inflammation in MASLD, and metformin ameliorates hepatic steatosis through both AMPK-dependent and AMPK-independent autophagy pathways. AMPK promotes lipophagy by suppressing mTORC1, upregulating Beclin1 and LC3-II, downregulating p62, and facilitating TFEB nuclear translocation (Inokuchi-Shimizu et al., 2014; Park et al., 2023; Li Z. et al., 2025). Concurrently, metformin induces autophagy through AMPK-independent mechanisms, including STAT3 downregulation and SIRT1-mediated or Parkin-mediated mitophagy (Song et al., 2015; Song et al., 2016; Li Y. L. et al., 2019). Based on our findings, we hypothesize that metformin-mediated Gpd2 inhibition alters intracellular energy status and redox balance, thereby indirectly modulating AMPK activity and initiating downstream signaling cascades, including mTORC1 suppression, ULK1 activation, and TFEB nuclear translocation, ultimately inducing autophagy. This proposed “Gpd2–AMPK–autophagy” axis remains a hypothesis awaiting experimental validation. This structural insight is further supported by the physiological distribution of metformin and the membrane localization of Gpd2: the liver is the principal site of metformin accumulation (Wilcock and Bailey, 1994), where hepatic concentrations are several-fold higher than plasma levels (He and Wondisford, 2015), and metformin accumulates in the mitochondrial matrix, while Gpd2 is located on the outer surface of the inner mitochondrial membrane, structurally positioned for potential direct interaction (Wang L. et al., 2024).

Apigenin, the natural flavonoid, which is highly abundant in celery, citrus, parsley, and chamomile, has a low toxicity, non-mutagenic, and safe safety profile (Wang C. et al., 2024), and NHANES data show that dietary apigenin intake is associated with a lower risk of MASLD (Wang C. et al., 2025). The ameliorative effects of apigenin have been attributed to MASH pathology to different indirect mechanisms, such as PPARα/γ/ activation (Shi et al., 2015), Keap1–Nrf2 pathway regulation (Yang et al., 2018), NLRP3 inflammasome suppression (Meng et al., 2023), and Rac1 (Hsu et al., 2025) and AMPK/SREBP signaling regulation (Lu et al., 2019). Nevertheless, none of the previous works has established a direct protein target that causes these pleiotropic effects. Our study is the first to computationally predict that apigenin may interact with Cybb/NOX2. This predicted affinity suggests a shift in the action mode of apigenin from indirect pathway modulation to potential direct interaction with the oxidative stress core hub.

Therefore, based on these dual-target findings, we additionally suggest a combination therapy using metformin and apigenin. Our *in vivo* experimental validations support this approach, demonstrating its superior efficacy in ameliorating MASH pathology compared to either monotherapy alone. This combination embodies an innovative multi-target treatment concept: metformin is predicted to primarily bind to Gpd2, thereby potentially intervening in lipotoxicity-driven oxidative stress at its metabolic origin, while apigenin is predicted to interact with Cybb/NOX2, potentially modulating inflammation and ROS generation at the enzymatic source.

Compared to the two FDA-approved drugs for MASH treatment in 2024–2025, resmetirom (Keam, 2024) and semaglutide (Bansal et al., 2026), this combination offers several potential advantages: wide availability, lower cost, well-established safety data from decades of clinical use, oral administration (versus injectable semaglutide), and simultaneous improvement in MASH pathology and associated metabolic parameters. However, it should be noted that although therapeutic efficacy was observed in our animal model, the low oral bioavailability of apigenin and significant species differences necessitate future improvements in formulation strategies, such as nano-delivery systems or phospholipid complexes, to enhance bioavailability, along with more systematic preclinical and clinical studies to validate its potential translational value.

Several limitations warrant consideration. First, a key limitation of the present study is that the differential expression of the identified hub genes (Gpd2, Cybb, Clec12a, Ifi211, and Tifab) has not yet been experimentally validated in our MASH model tissues by qPCR, Western blot, or immunohistochemistry. Although these genes were identified through robust cross-cohort bioinformatics mining and machine-learning prioritization, their protein-level expression and cellular localization in MASH liver remain to be confirmed. Second, although molecular dynamics simulations provided structural evidence for stable binding, further experimental confirmation using cell-based target engagement assays [e.g., surface plasmon resonance (Wang et al., 2026), cellular thermal shift assay (Tu et al., 2023), or pull-down assay] would strengthen the conclusion that these compounds act as direct protein inhibitors. Third, the optimal dosage, therapeutic window, and pharmacokinetic interactions between metformin and apigenin remain to be systematically investigated in larger animal models and validated in human primary hepatocytes before progression to clinical trials. Finally, the dynamic changes in this gene signature across various disease stages (from simple steatosis to advanced fibrosis) should then be further longitudinally characterized to comprehensively define its possible use as a disease monitoring and predictive biomarker of therapeutic responses.

## 5 Conclusion

To sum up, in this study, we use the integrative multi-omics analysis to reveal a five-gene signature that mechanistically connects hepatic dysregulation of lipids and inflammatory networks in MASH. Through the translation of such genomic findings into viable therapeutic targets, we show that repositioned metformin and apigenin (targeting Gpd2 and Cybb, respectively) have better preclinical activity than monotherapy. This approach will address essential unmet requirements in the MASH management by providing a therapeutic alternative that is readily available, orally administered, and less expensive. Significantly, co-targeting multiple disease-driving pathways simultaneously has the potential to improve response rates, which are suboptimal in single-agent therapy and achieve better therapeutic outcomes in the diverse MASH population. These results justify the effectiveness of transcriptome-based network pharmacology in increasing the pace of clinical translation of multi-targeted regimens in complex metabolic-inflammatory illnesses.

## Data Availability

The raw data have been deposited in the CNGB Nucleotide Sequence Archive (CNSA) under accession number CNP0008322.

## Glossary

MASH: metabolic dysfunction-associated steatohepatitis
MASLD/MAFLD: metabolic dysfunction-associated steatotic liver disease
HCC: hepatocellular carcinoma
FDA: Food and Drug Administration
ALT: alanine aminotransferase
AST: aspartate aminotransferase
TG: triglycerides
TCHO: total cholesterol
HDL: high-density lipoprotein
LDL: low-density lipoprotein
PCA: principal component analysis
GO: Gene Ontology
KEGG: Kyoto Encyclopedia of Genes and Genomes
WGCNA: weighted gene co-expression network analysis
ROC: receiver operating characteristic
GSEA: Gene Set Enrichment Analysis
MR: Mendelian randomization
IVs: instrumental variables
IVW: inverse variance weighted
MD: molecular dynamics
RMSD: root mean square deviation
RMSF: root mean square fluctuation
Rg: radius of gyration
SASA: solvent-accessible surface area
Gpd2: mitochondrial glycerol-3-phosphate dehydrogenase 2
G3P: glycerol-3-phosphate
DHAP: dihydroxyacetone phosphate
DAG: diacylglycerol
PC: phosphatidylcholine
PE: phosphatidylethanolamine
Cybb: cytochrome b-245 beta chain
NOX2: NADPH oxidase 2
ROS: reactive oxygen species
sNOX2-dp: serum soluble NOX2-derived peptide (sNOX2-dp)
NAS: NAFLD activity score
PS: phosphatidylserine
Clec12a: C-type lectin domain family 12 member A
DAMPs: damage-associated molecular patterns
Ifi211: interferon-inducible protein 211
MNDA: myeloid nuclear differentiation antigen
TIFAB: tumor necrosis factor receptor-associated factor-interacting protein with forkhead-associated domain B
T2MD: type 2 diabetes mellitus
AMPK: AMP-activated protein kinase
