# Supplementary material for: Repurposed metformin–apigenin combination therapy for metabolic dysfunction-associated steatohepatitis: from integrative multi-omics discovery to in vivo validation
Source: Front Pharmacol. 2026 Sep 4;17:1857676. doi: 10.3389/fphar.2026.1857676 (PMC13586655; doi:10.3389/fphar.2026.1857676)
Supplement: Supplementary file 1 [file Supplementaryfile2.docx]

Supplementary Fig.S1. Immune infiltration analysis and Correlations of hub genes in human liver tissues.

(A) Differential boxplots of immune cells from CIBERSORT results. (B) Heat map of correlation of biomarker genes and differential immune cells. (C) Correlations of hub genes in human liver tissues. *p <0.05, **p <0.01, ***p <0.001, ****p <0.0001. Clec12a, C-type lectin domain family 12 member A ;Cybb, Cytochrome b-245 beta chain; Gpd2, Glycerol-3-phosphate dehydrogenase 2; Ifi211, Interferon-activated gene 211; Tifab, TRAF-interacting protein with forkhead-associated domain B. MNDA, Myeloid nuclear differentiation antigen .

Supplementary Fig.S2. Expression annalysis of CYBB and GPD2 across different groups in the human GSE61260 dataset

CYBB showed no differential expression between healthy controls and obese individuals. However, CYBB was significantly elevated in both MAFL and MASH compared with controls, with no further increase between MAFL and MASH (P < 0.05). In contrast, GPD2 showed no significant differences among healthy controls, obese individuals, and MAFL patients, but was significantly elevated in MASH compared with all other groups (P < 0.05). *p <0.05, **p <0.01, ***p <0.001, ****p <0.0001. MAFL，Metabolic dysfunction-Associated Fatty Liver; MASH，Metabolic dysfunction-Associated Steatohepatitis.

Supplementary Fig.S3. Sensitivity and heterogeneity analysis of MR.

(A-D )Forest and (E-H )scatter plots of Clec12a,Gpd2，MNDA，Tifab. (I-L) Funnel plots of Clec12a,Gpd2，MNDA，Tifab. (M-P) leave-one-out analysis of Clec12a,Gpd2，MNDA，Tifab. Clec12a, C-type lectin domain family 12 member A ; Gpd2, Glycerol-3-phosphate dehydrogenase 2; Ifi211, Interferon-activated gene 211; Tifab, TRAF-interacting protein with forkhead-associated domain B. MNDA, Myeloid nuclear differentiation antigen .

Supplementary Fig.S4. Drug screening and molecular docking results of Clec12a,Ifi211,and Tifab.

(A,B) Global and Local molecular docking of Venetoclax-Clec12a. (C,D) Global and Local molecular docking of Upadacitinib-Ifi211. (E,F) Global and Local molecular docking of Metronidazole-Tifab. Clec12a, C-type lectin domain family 12 member A ;Ifi211, Interferon-activated gene 211; Tifab, TRAF-interacting protein with forkhead-associated domain B.
